# HLA-B*58:01 allele is strongly associated with allopurinol-induced severe cutaneous adverse reactions in a Thai population

**DOI:** 10.1186/2045-7022-4-S3-P120

**Published:** 2014-07-18

**Authors:** Thawinee Jantararoungtong, Ticha Rerkpattanapipat, Santirat Prommas, Napatrupron Koomdee, Siwalee Santon, Montri Chammanphol, Apichaya Puangpetch, Chonlaphat Sukasem

**Affiliations:** 1 Ramathibodi Hospital, Laboratory for Pharmacogenomics, Thailand; 2 Ramathibodi Hospital, Medicine Department, Thailand

## Background

Allopurinol has been reported as the most frequent causes of SCARs (severe cutaneous adverse reactions) in Thailand. Recent publications have shown that HLA-B*58:01 allele is a strong marker for allopurinol-induced SJS/TEN (Stevens-Johnson syndrome/toxic epidermal necrolysis). The aim of this study was to clarify the association of allopurinol-induced SCARs with the HLA-B*58:01 allele in Thai patients.

## Method

To investigate this relationship, we performed PCR-SSOP (sequence specific oligonucleotide probe) on allopurinol-tolerant controls (n=56) and patients affected by allopurinol-induced SCARs (n=14). Among of allopurinol-induced SCARs, including 3 patients with allopurinol-induced DRESS (drug reaction with eosinophilia and systemic symptoms), 9 patients with SJS/TEN and 2 patients with MPE (maculo-papular exanthema) were included. The presence of HLA-B*58:01 allele were genotyped by PCR-SSOP method at Laboratory for Pharmacogenomics, Ramathibodi Hospital.

## Results

Of the 14 patients with allopurinol-induced SCARs, 13 (92.8%) patients (3 DRESS, 9 SJS/TEN and one severe MPE) had HLA-B*58:01 while only 6 (10.71%) of the allopurinol-tolerant controls had this allele. The risk of allopurinol-induced SCARs was significantly higher in the patients with HLA-B*58:01 with an OR (odd ratios) of 108.33 (95% CI 11.96-980.82, p<10-6). When compared with normal population, HLA-B*58:01 was associated with a higher risk of SCARs, both DRESS (OR: 80, 95%CI 3.42-372.87) and SJS/TEN (OR: 217.26, 95%CI 12.41-925.35).

## Conclusion

In this study we confirmed that HLA-B*58:01 allele is strongly associated with allopurinol-induced SCARs in Thai population. Therefore, screening tests for HLA-B*58:01 allele in patients who will be treated with allopurinol will be clinically helpful in preventing the risk of developing SCARs.

